# Acute cardiovascular changes following heat exposure during simulated shipboard firefighting

**DOI:** 10.1113/EP092779

**Published:** 2026-03-09

**Authors:** Daniel K. Sweet, Elizabeth M. Lavoie, Hayden W. Hess, Stuart Inglis, Brian Monaco, J. Luke Pryor, Steven E. Reis, David Hostler

**Affiliations:** ^1^ Center for Research and Education in Special Environments, Exercise and Nutrition Sciences University at Buffalo Buffalo New York USA; ^2^ Department of Pathology and Anatomical Sciences University at Buffalo Buffalo New York USA; ^3^ Department of Emergency Medicine University at Buffalo Buffalo New York USA; ^4^ Department of Medicine University of Pittsburgh Pittsburgh Pennsylvania USA

**Keywords:** central blood pressure, echocardiography, microvascular reactivity

## Abstract

Like structural firefighting, shipboard firefighting requires extreme exertion. However, shipboard firefighting may be a unique cardiovascular stress as most sailors lack extensive firefighting experience and may complete significant work before reaching the fire scene. Several indices of acute cardiovascular impairment have been associated with the high prevalence of sudden cardiac death among structural firefighters. However, acute cardiovascular responses to shipboard firefighting have yet to be described. The aim of this study was to investigate cardiac, macrovascular and microvascular responses to simulated shipboard firefighting. Nineteen participants donned protective equipment before completing the simulated shipboard firefighting protocol (SBFFP), which included lifting, striking and pulling tasks repeated until a stopping criterion was met (maximum heart rate, volitional fatigue, breathing air supply depleted). Echocardiography, aortic and brachial blood pressure, subendocardial viability ratio (SEVR), and reactive hyperaemia index were measured before, after and 1 h after the protocol. Immediately after SBFFP, left ventricular end diastolic and systolic diameter, brachial systolic blood pressure, SEVR, and the reactive hyperaemia index decreased (*P* ≤ 0.0121), while ventricular fractional shortening increased (*P* < 0.0001). All of these metrics returned to baseline after 1 h (*P* ≥ 0.0699), except SEVR, which remained decreased (*P* = 0.0400). There were no changes in aortic blood pressures (*P* ≥ 0.0671). These data represent the cardiovascular strain and subsequent impairment expected after shipboard firefighting operations, and may be mechanisms contributing to acutely increased risk of cardiac events after firefighting.

## Abstract (highlights)

- **What is the central question of this study?**
  How do metrics of cardiac, macrovascular and microvascular function respond to simulated shipboard firefighting and do these responses mirror those of structural firefighting?
- **What is the main finding and its importance?**
  Cardiac and microvascular impairments after shipboard firefighting provide evidence for potential myocardial ischaemia, which could acutely increase the probability of a cardiac event.

## 1 INTRODUCTION

Shipboard fires present great risk to sailors’ safety and can overwhelm medical resources, leading to compromised fleet readiness and the potential for a mass casualty event (Wickard et al., 2025). Recent cases, such as the loss of the USS Bonhomme Richard in 2020, have necessitated updated research describing physiological responses to shipboard firefighting, including data describing sailors’ cardiovascular strain and subsequent responses. Shipboard firefighting is unique because crew members with minimal firefighter experience are required to respond to fire emergencies. Before engaging in firefighting work, shipboard firefighters must first carry fire suppressant and equipment to the scene rather than arriving at the scene in a vehicle with the necessary equipment, as is usually the case in structural firefighting. This decreased familiarity with firefighting tasks and additional strain preceding the firefight may result in excessive cardiovascular strain. While reports of heart rate (HR) and core body temperature responses to shipboard firefighting were reported to be similar to structural firefighting (Bennett et al., 1995), more nuanced metrics of cardiovascular strain and function when donning modern protective garments have not been produced.

Sudden cardiac death has been the predominant cause of on‐duty fatalities among structural firefighters (Kales et al., 2007). In 2023, 36 firefighters died within 24 h of duty from cardiac events (Campbell et al., 2024). As a population, firefighters have an elevated risk of cardiovascular disease, evidenced by high prevalence of hypertension, dyslipidaemia and obesity (Bode et al., 2021). Firefighting operations elicit intense exertion, heat stress and psychological strain, which present a severe cardiovascular challenge (Smith et al., 2016). While there have not been reports of sudden cardiac death during or after shipboard firefighting, there is evidence of cardiovascular disease risk among US Navy personnel. A study of a submarine crew reported an obesity prevalence of 62%, and 30% of the submariners with obesity had insulin resistance (Gasier et al., 2016).

Several metrics of cardiovascular function have been employed to examine mechanisms that may be related to firefighters’ cardiac events. Echocardiography has been used to assess the strain and function of the myocardium following firefighting. In two studies investigating responses to a 3‐h live fire training, ventricular function was impaired, evidenced by reduced fractional shortening (Fernhall et al., 2012) and ejection fraction (Hibner et al., 2022).

Systolic blood pressure may increase during firefighting tasks (Bugajska et al., 2007), but heat stress and subsequent cutaneous vasodilation consistently result in transient relative hypotension during recovery (Burgess et al., 2012; Horn et al., 2011; Hostler et al., 2016). Non‐invasive measurement of aortic blood pressure via radial artery applanation tonometry has been validated (Pauca et al., 2001; Sharman et al., 2006) and is a better predictor of future cardiovascular events than brachial blood pressure (McEniery et al., 2014). In response to firefighting, there have been reports of no change or a modest increase in aortic blood pressures immediately after firefighting, followed by a return to baseline or aortic hypotension during recovery (Fahs et al., 2011; Horn et al., 2011; Santos et al., 2023). Applanation tonometry also allows for measurement of the subendocardial viability ratio (SEVR), which is a ratio of the aortic diastolic and systolic pressure time intervals and represents the proportion of myocardial oxygen delivery, relative to demand (Crilly et al., 2007). This ratio has been shown to decrease after firefighting, indicating a lower myocardial reserve and potentially greater vulnerability to ischaemia (Horn et al., 2011).

The reactive hyperaemia index (RHI) is a measurement of microvascular function whereby a higher RHI is associated with a lower risk of cardiac events (Matsuzawa et al., 2015). There have been reports of impaired (Andersen et al., 2017) and improved (Olafiranye et al., 2015) microvascular function following firefighting. These disparate results could be due to methodological differences, as the study reporting impaired RHI was a live fire extinction training exercise, while the study reporting improved RHI employed treadmill exercise while wearing firefighting garments.

Cumulatively, the data from structural firefighting describe a scenario of cardiovascular vulnerability characterized by increased myocardial strain and impaired vascular function. None of these metrics of cardiovascular strain and function have been studied following shipboard firefighting. Therefore, the aim of this investigation was to evaluate microvascular, macrovascular and cardiac responses to simulated shipboard firefighting. Similar to structural firefighting, we hypothesize that shipboard firefighting tasks induce cardiovascular strain, indicated by decreased fractional shortening, RHI, SEVR and increased aortic blood pressure.

## 2 METHODS

### 2.1 Ethical approval

Before enrolling in the study, each participant provided written informed consent after the study protocols and risks were explained. The study conformed to the standards outlined in the *Declaration of Helsinki* and was approved by the university institutional review board (Study 00007237).

### 2.2 Study design

This was a cross‐sectional study including 18 males and one female consisting of three separate laboratory visits, each separated by at least 7 days. Before each visit, participants did not exercise or consume alcohol, caffeine or nicotine for 12 h. Participants were excluded if they had a history of cardiovascular disease, hypertension, pregnancy, use of medications which could affect physiological responses to heat or exercise, or a history of nicotine use. In order to be included in the study, participants had to score ‘probationary’ or better on at least one task (e.g., push‐ups, plank, cardiovascular endurance) of the 2023 US Navy Physical Readiness test, which was conducted during the first visit. After the health history screening and Physical Readiness test, participants completed a brief familiarization with the simulated shipboard firefighting protocol (SBFFP); the second visit consisted of fitness testing (e.g., maximum aerobic capacity, whole‐body strength, body composition). Waist circumference was measured by three‐dimensional optical imaging (SS20 3D Body Scanner, Size Stream, Cary, NC, USA). In the third visit, participants completed the SBFFP, with cardiovascular measures before, immediately after and 1 h after. As this is a secondary analysis, methods of the first two visits and SBFFP have been detailed previously (Sweet, Lavoie et al., 2025). Briefly, participants donned firefighting protective garments, helmet and self‐contained breathing apparatus, then completed an 80 m walk followed by carrying two 20 kg buckets up and down a flight of stairs before beginning a circuit of tasks that was repeated until a stopping criterion was met. The circuit included lifting and striking tasks completed in an environmental chamber set to 40°C and 40% relative humidity. These conditions create changes in heart rate and body temperature similar to those seen in previous field studies. An upper body push and pull task was completed in an adjacent temperate environment. These circuits were repeated until participants requested to stop, HR reached age‐predicted maximum (220 − age), or gas within the self‐contained breathing apparatus was depleted.

### 2.3 Echocardiography

Echocardiographic images were taken in two‐dimensional M‐mode (Vivid q, GE Healthcare, Chicago, IL, USA) through the parasternal short axis view at the mid‐papillary plane of the left ventricle. Left ventricular end systolic diameter (LVESD) and end diastolic diameter (LVEDD) measurements were taken in triplicate and averaged before calculating fractional shortening ($\mathrm{FS}\%\;=\frac{\mathrm{EDD}-\mathrm{ESD}}{\mathrm{EDD}}\;\times 100$). Echocardiography was performed with the participant in the seated position before, within 5 min after, and 1 h after the SBFFP. Echocardiography was performed by a single researcher with coefficients of variation of 7%, 9% and 1% for LVESD, LVEDD and fractional shortening, respectively.

### 2.4 Brachial and aortic and blood pressures

Brachial blood pressure was measured with an automated machine (Propaq CS, WelchAllyn, Skaneateles Falls, NY, USA) before aortic blood pressure was measured via radial artery applanation tonometry (SphygmoCor EM3, Cardiex USA, Naperville, IL, USA) whereby a generalized transformation function is applied to the radial pulse wave measurement to obtain an aortic pulse wave, yielding blood pressures and SEVR (Crilly et al., 2007; Pauca et al., 2001; Sharman et al., 2006). The rate–pressure product was calculated as brachial systolic blood pressure × HR. Brachial and aortic blood pressures were measured with the participant in the seated position before, within 10 min after and 1 h after the SBFFP.

### 2.5 Microvascular function

To measure RHI, peripheral artery tonometry (EndoPAT 2000; ZOLL Itamar, Ltd, Atlanta, GA, USA) was used to measure pulse amplitude at each index finger throughout a 15 min test consisting of 5 min baseline measurement, 5 min blood flow occlusion of the right arm, and 5 min of recovery measurements. The left arm tonometry data serve as a control for endothelium‐independent variations. The tonometry software calculated the RHI by dividing the ratio of mean pulse amplitude before and after the 5‐min occlusion in the right arm by the same ratio in the left arm. A greater hyperaemic response in the right arm following occlusion increases the RHI value indicating healthy vascular function. Accurate RHI measurement requires a 15 min rest before testing begins. Therefore, RHI measurements were performed before, 15 min after and 75 min after the SBFFP.

### 2.6 HR and thermoregulatory data

To measure core body temperature, participants swallowed a telemetry pill (eCelsius Performance, Hérouville Saint‐Clair, France) 6–8 h prior to the protocol (*n* = 14) or inserted it rectally immediately before (*n* = 5) the protocol. Core body temperature, mean skin temperature (Ramanathan, 1964) (iButton, Maxim Integrated, San Jose, CA, USA), core‐to‐skin temperature gradient, and HR (Polar Electro Inc., Bethpage, NY, USA) were measured immediately following completion of echocardiography before, after and 1 h after the SBFFP. Additionally, HR was also monitored throughout SBFFP and peak HR was recorded as percentage age‐predicted maximum (% HR_Max_) (Tanaka et al., 2001).

### 2.7 Statistical analyses

After all data were checked for normality (Shapiro–Wilk test) and equal variance (Levene's test), and separate one‐way repeated‐measures analysis of variance tests (ANOVAs) assessed changes in cardiovascular measures before and after the SBFFP. If an ANOVA revealed a significant time effect, Tukey's multiple comparisons test compared individual time points.

An additional exploratory analysis was conducted to examine the effect of cardiovascular strain on subsequent responses. First, participants were grouped into tertiles of peak HR during SBFFP (low, <96.0% HR_Max_ [*n* = 6]; moderate, 96.0–101.7% HR_Max_ [*n* = 7]; and high, >101.7% HR_Max_ [*n* = 6]). Then, for the cardiovascular variables indicating cardiovascular impairment after SBFFP, change scores were calculated from baseline. Finally, separate two‐way (time × tertile) repeated measures ANOVAs assessed interactions between peak HR during SBFFP and the impaired cardiovascular variables. All analyses were conducted in GraphPad Prism (version 10.0.2, GraphPad Software, Boston, MA, USA) with significance set to *P* < 0.05. Data are reported as means ± SD.

## 3 RESULTS

Participants were 24 ± 5 years of age with 25.6 ± 2.9 kg m^−2^ BMI, 89 ± 8 cm waist circumference, 3.62 ± 0.71 L min^−1^ maximum oxygen consumption, and reported 420 ± 360 weekly min of physical activity. All participants scored ‘probationary’ or better on at least one task within the Physical Readiness Test. Cumulative Physical Readiness Test Scores were as follows: six participants scored ‘probationary’ or worse, one ‘satisfactory’, ten ‘good’, and two ‘excellent’. During the Bruce protocol to measure maximum oxygen consumption, participants achieved peak respiratory exchange ratio, minute ventilation and HR of 1.17 ± 0.08, 139.3 ± 31.4 L min^−1^ and 191 ± 11 bpm, respectively. Before meeting stopping criteria, participants were in the SBFFP for 24 ± 9 min. HR, core body temperature and mean skin temperature all increased from baseline immediately after SBFFP and did not fully recover to baseline values after 1 h (*P* ≤ 0.0006, Table 1). The core‐to‐skin temperature gradient decreased immediately after SBFFP and also did not fully recover to baseline values after 1 h (*P* ≤ 0.0176, Table 1). Detailed physiological responses during the SBFFP have been published previously (Sweet, Lavoie et al., 2025).

**TABLE 1:** Cardiovascular and thermoregulatory responses to simulated shipboard firefighting (*n* = 19).

					*P*		
		Pre	Post	1 h post	ANOVA	Pre–post	Pre–1 h post
HR (bpm)		68 ± 8	117 ± 11^a^	82 ± 10^a^	<0.0001	<0.0001	<0.0001
Core body temperature (°C)		37.0 ± 0.2	38.1 ± 0.5^a^	37.2 ± 0.3^a^	<0.0001	<0.0001	0.0006
Mean skin temperature (°C)		30.9 ± 0.8	35.6 ± 0.8^a^	31.8 ± 0.7^a^	<0.0001	<0.0001	<0.0001
Core‐to‐skin temperature gradient (°C)		6.0 ± 0.8	2.6 ± 0.5^a^	5.5 ± 0.7^a^	<0.0001	<0.0001	0.018
Rate pressure product (bpm mmHg)		8278 ± 1125	14608 ± 1597^a^	9684 ± 1445^a^	<0.0001	<0.0001	<0.0001
Subendocardial viability ratio (%)		212 ± 47	108 ± 16^a^	162 ± 20^a^	<0.0001	<0.0001	0.040
Brachial blood pressures (mmHg)	Systolic	122 ± 11	125 ± 15	118 ± 10	0.129	—	—
	Diastolic	71 ± 6	66 ± 6^a^	66 ± 7	0.014	0.028	0.070
	Mean	87 ± 6	85 ± 8	82 ± 7	0.055	—	—
	Pulse pressure	51 ± 12	59 ± 13	52 ± 9	0.025	0.056	0.927
Aortic blood pressures (mmHg)	Systolic	103 ± 8	102 ± 11	98 ± 7	0.108	—	—
	Diastolic	72 ± 6	70 ± 7	68 ± 7	0.091	—	—
	Mean	87 ± 6	85 ± 8	82 ± 7	0.067	—	—
	Pulse pressure	31 ± 7	32 ± 7	30 ± 5	0.553	—	—

^a^ Difference from baseline (Pre).

Repeated‐measures ANOVA revealed an effect of time on the rate–pressure product, SEVR, brachial diastolic blood pressure and brachial pulse pressure (*P* ≤ 0.0245, Table 1). The rate–pressure product increased (*P* < 0.0001) while SEVR decreased (*P* < 0.0001) immediately after SBFFP and neither returned to baseline after 1 h (*P* ≤ 0.0020). Brachial diastolic blood pressure was decreased after SBFFP compared to baseline (*P* = 0.0272), but *post hoc* tests did not reveal any differences between time points in brachial pulse pressure. There were no differences across time for brachial systolic and pulse pressure nor any of the aortic blood pressures (*P* ≥ 0.0551, Table 1).

**FIGURE 1:**
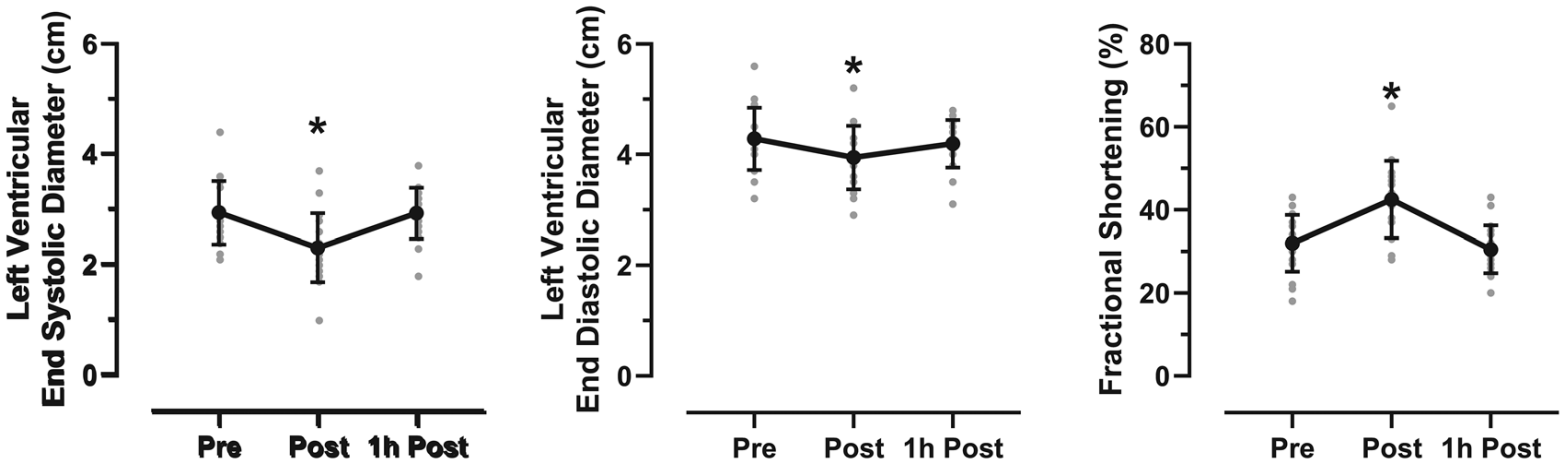
Left ventricular responses to simulated shipboard firefighting (*n* = 19). Immediately after firefighting (Post), end systolic diameter and end diastolic diameter were reduced (*P* ≤ 0.012) while fractional shortening increased (*P* < 0.0001). All returned to baseline within 1 h after firefighting.

All of the echocardiographic data changed over time (*P* ≤ 0.0097, Figure 1). Both LVESD and LVEDD were decreased (*P* ≤ 0.0121) while fractional shortening was increased (*P *< 0.0001), after SBFFP. All three variables had recovered 1 h after SBFFP (*P* ≥ 0.4623).

**FIGURE 2:**
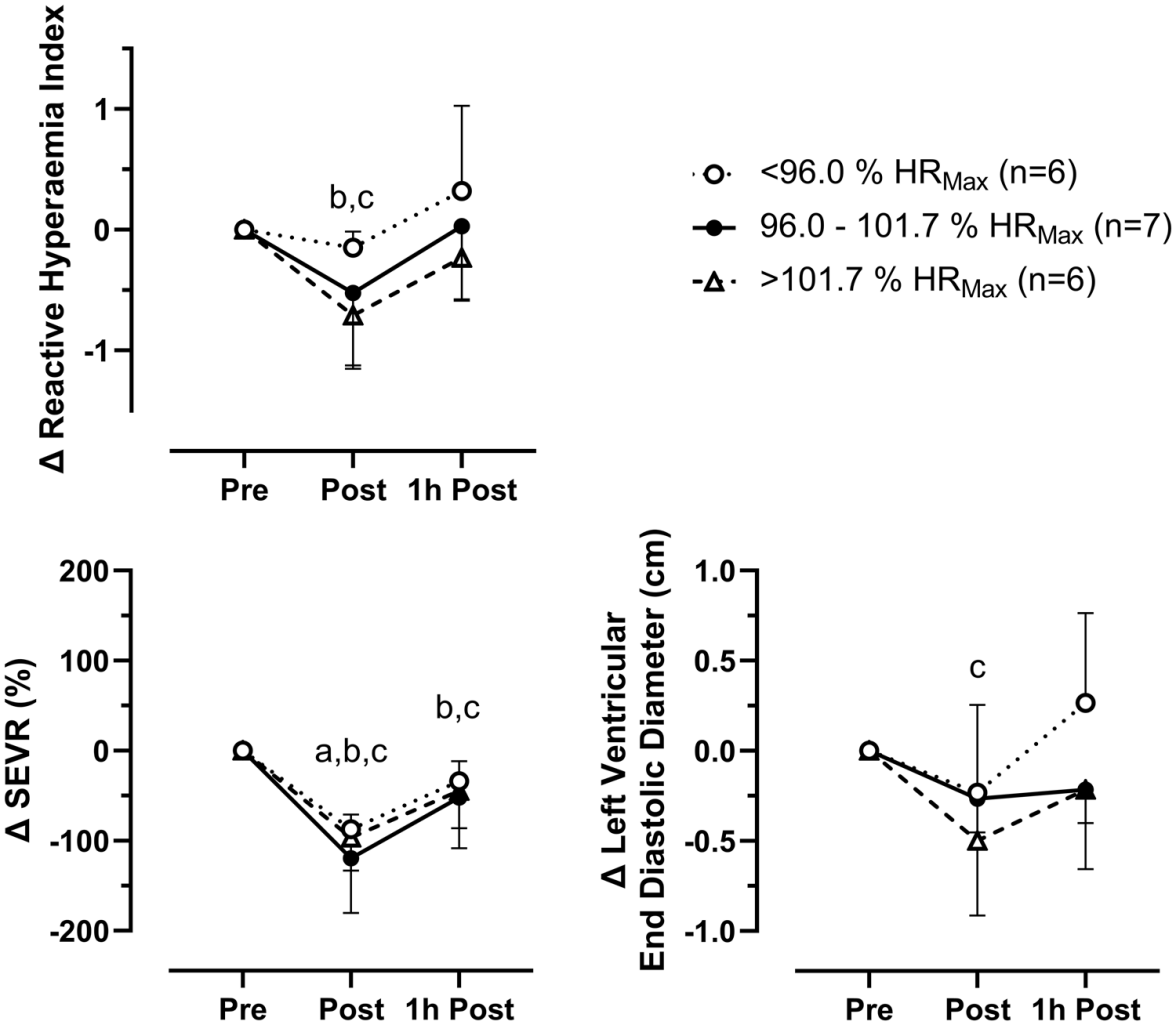
Microvascular impairment following simulated shipboard firefighting (*n* = 19). Immediately after firefighting (Post), reactive hyperaemia index was reduced (*P* = 0.002) to the threshold for clinical microvascular impairment (dotted line; RHI ≤ 1.35). Microvascular function returned to baseline within 1 h after firefighting (*P* = 0.957).

Microvascular function changed over time (*P* = 0.0003, Figure 2) such that RHI was decreased immediately after SBFFP (*P* = 0.0018) and recovered to baseline values 1 h after (*P* = 0.9565).

**FIGURE 3:**
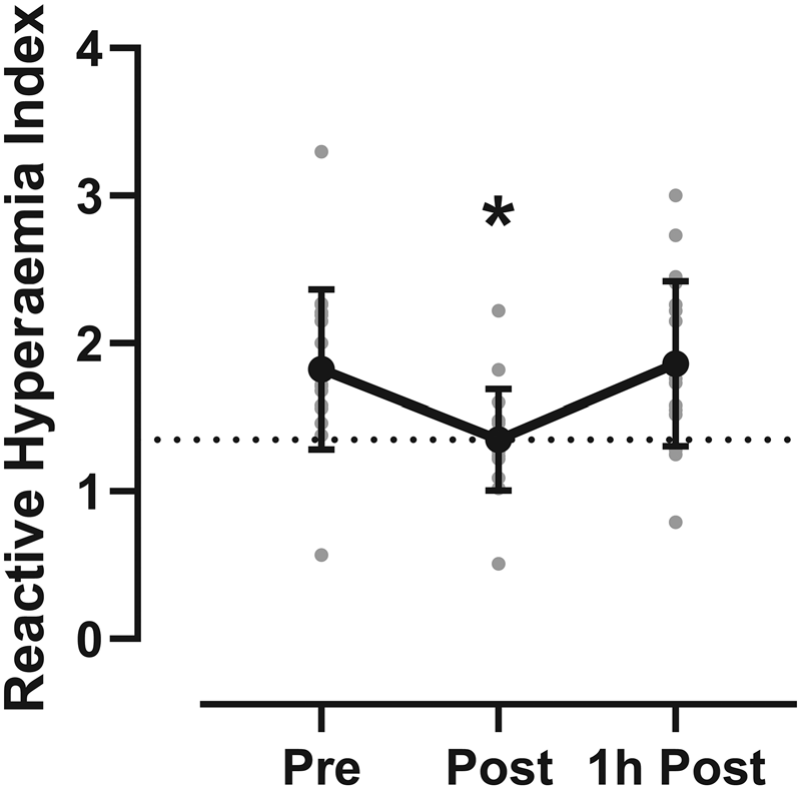
Cardiovascular impairment as a function of peak HR achieved during simulated shipboard firefighting. Reactive hyperaemia index: only participants who achieved a HR > 96% of age‐predicted maximum (HR_Max_) had microvascular impairment (*P* ≤ 0.036) immediately after the protocol (Post). SEVR: Post, all groups had decreased coronary oxygen supply, relative to demand (*P* ≤ 0.0005), but only those with <96% HR_Max_ recovered after 1 h (*P* ≤ 0.026). End diastolic diameter: Post, only those with >101.7% HR_Max_ had decreased end diastolic diameter. ^a^<96.0% HR_Max_ group different from Pre. ^b^96.0–101.7% HR_Max_ group different from Pre. ^c^>101.7% HR_Max_ group different from Pre.

The peak HR tertile analysis was applied to RHI, SEVR and LVEDD change scores. Table 2 details characteristics of the participants in each tertile. Each group's peak HR was different from the others (*P* ≤ 0.0027) but the groups did not differ by age, BMI, maximum oxygen consumption or time in SBFFP (*P* ≥ 0.3492). At baseline, RHI (low, 1.62 ± 0.60; moderate, 1.91 ± 0.64; high, 1.93 ± 0.35; *P* ≥ 0.6208), SEVR (low, 189% ± 21%; moderate, 232% ± 56%; high, 204% ± 44%; *P* ≥ 0.3267) and LVEDD (low, 4.1 ± 0.6 cm; moderate, 4.5 ± 0.6 cm; high, 4.2 ± 0.5 cm; *P* ≥ 0.4585) did not differ between tertiles but there was an effect of time on RHI, SEVR and LVEDD change scores (*P* ≤ 0.0203, Figure 3). The moderate and high tertile groups had decreased RHI immediately after SBFFP (*P* ≤ 0.0356), while the low tertile had unchanged RHI throughout (*P* ≥ 0.269). All tertiles had decreased SEVR immediately after SBFFP (*P* ≤ 0.0005), but only the low tertile recovered to baseline values after 1 h (*P* ≤ 0.0261). Immediately after SBFFP, only the high tertile had decreased LVEDD, which recovered to baseline values 1 h after (*P* = 0.0062).

**TABLE 2:** Characteristics of participants by tertiles of peak heart rate (% age‐predicted maximum; % HR_Max_) during the simulated SBFFP.

				*P*		
	<96.0% HR_Max_ (low; *n* = 6)	96.0–101.7% HR_Max_ (mod; *n* = 7)	>101.7% HR_Max_ (high; *n* = 6)	Low vs. mod	Low vs. high	Mod vs. high
Age (years)	27 ± 6	23 ± 5	23 ± 3	0.324	0.349	>0.999
BMI (kg m^−2^)	25.9 ± 3.6	24 ± 1.8	26.6 ± 8.8	0.904	0.973	0.788
${\dot{V}}_{O_{2}\max}$ (L min^−1^)	3.61 ± 1.22	3.71 ± 0.83	3.62 ± 0.71	0.980	>0.999	0.984
Time in SBFFP (s)	1207 ± 550	1510 ± 544	1565 ± 460	0.556	0.476	0.980
Peak HR in SBFFP (% max)	93.4 ± 1.5^b^	99.8 ± 1.5^a^, ^b^	103 ± 1.0^a^	<0.0001	<0.0001	0.003

^a^ Difference from Low HR_Max_ group.

^b^ Difference from Mod HR_Max_ group.

## 4 DISCUSSION

This was the first investigation of cardiovascular changes after simulated shipboard firefighting. We have previously reported that this protocol resulted in hyperthermia and near‐maximal HR (Sweet, Lavoie et al., 2025), similar to older reports of shipboard (Bennett et al., 1995) and structural firefighting (Al‐Zaiti et al., 2015; Hostler et al., 2016; Schlader et al., 2017). In line with our hypothesis, the cardiovascular impairment observed in the present report was mostly similar to previous reports from structural firefighting studies, with some notable exceptions.

In the structural firefighting literature, there has been consistent reporting of transient hypotension following firefighting activities (Burgess et al., 2012; Horn et al., 2011; Hostler et al., 2016). These previous studies all reported no change in systolic blood pressure immediately after firefighting, with modest hypotension observed 10–40 min after. In this study, the 1 h gap in data collection time points after SBFFP likely included this period of systolic hypotension during recovery, but the diastolic hypotension that we observed immediately after SBFFP is similar to previous reports (Horn et al., 2011; Hostler et al., 2016) and is likely a result of vasodilation, secondary to hyperthermia (Crandall, 2008).

We did not observe changes in aortic blood pressures. Similarly, aortic blood pressure was unchanged after 18 min of firefighting activities within a population similar to that of the present report (Horn et al., 2011). Reports of increased aortic blood pressures, however, have been documented after a 3 h training exercise (Fahs et al., 2011) in which participants experienced greater hyperthermia than the present report (∼38.9°C) and within a middle‐aged and overweight population (Lefferts et al., 2021). Therefore, aortic blood pressure responses to firefighting seem dependent on both the population (i.e., baseline cardiovascular disease risk factors) and duration or thermal strain of the work.

Contrary to previous reports of decreased left ventricular contractile performance following structural firefighting (Fernhall et al., 2012; Hibner et al., 2022), we documented increased fractional shortening following simulated shipboard firefighting. In the previous studies, echocardiography was performed 10–30 min after firefighting, at which point systolic parameters had returned to baseline, but diastolic parameters were decreased. In the present report, echocardiography was performed within 5 min of protocol stop, at which point LVESD and LVEDD were both decreased from baseline. So, it is likely that echocardiography was measured in a greater inotropic state, evidenced by tachycardia (Mulieri et al., 1992), compared to previous structural firefighting reports. This presents a sequence in which high sympathetic tone seems to preserve cardiac function during firefighting, but it may then deteriorate during sympathetic withdrawal during recovery. Indeed, firefighters’ risk of sudden cardiac death remains 2.2 to 10.5 times elevated in the hours following emergency response (Kales et al., 2007). Tachycardia immediately after SBFFP also limited diastolic filling time, contributing to decreased LVEDD. Diastolic filling could have also been limited by decreased venous return resulting from the vasodilatory response to hyperthermia (i.e., decreased diastolic blood pressure). Impairments in cardiac diastolic function may be an indication of ischaemic heart failure (Grossman, 1990).

Similar to structural firefighting responses (Horn et al., 2011), SEVR was reduced immediately after SBFFP, indicating less coronary blood flow relative to the increased myocardial oxygen demand immediately following the protocol, potentially rendering the heart more susceptible to ischaemia (Crilly et al., 2007). Concurrently, the rate pressure product was elevated, implying increased myocardial work and oxygen consumption after SBFFP (White, 1998). It is notable that neither SEVR nor the rate pressure product recovered to baseline 1 h after SBFFP, demonstrating the prolonged period of increased myocardial demand and ischaemic vulnerability after firefighting.

At rest, RHI has been associated with coronary blood flow (Bonetti et al., 2004) and coronary microvascular function (Di Serafino et al., 2021; Nardone et al., 2019; Toya et al., 2025). While the relationship between RHI and coronary microvascular function has not been examined after exercise or heat stress, decreased RHI observed 15–30 min after SBFFP suggests that coronary blood flow may be compromised during recovery after intense firefighting activity. At this time point, RHI decreased to 1.34 ± 0.34. Clinically, RHI < 1.35 indicates coronary endothelial dysfunction (Bonetti et al., 2004). To accommodate increased cardiac demand, coronary oxygenation must be augmented primarily by vasodilation (Goodwill et al., 2017). Therefore, after firefighting when the myocardium may be vulnerable to ischaemia, coronary endothelial function is critical.

The microvascular impairment observed here is similar to a report following live fire training (Andersen et al., 2017) but contrary to the lack of change reported after continuous treadmill exercise with heat stress (Olafiranye et al., 2015). Previous work from our laboratory (Sweet, Patterson et al., 2025) and others (Johnson et al., 2012; Landers‐Ramos et al., 2025) has provided evidence that exercise intensity may influence post‐exercise vascular function. These disparate results after firefighting and exertional heat stress may be related to thermoregulatory strain, exercise type, volume, and intensity or total muscle mass used during the exertion (i.e., full firefighting simulation/training versus treadmill walking in firefighting garments).

We conducted an exploratory analysis to test the hypothesis that the cardiovascular impairment that we observed may have been dependent on the magnitude of cardiovascular strain incurred during SBFFP (Figure 3). Regardless of peak HR during SBFFP, SEVR was decreased immediately after SBFFP, but SEVR only recovered after 1 h in participants who did not exceed 96% HR_Max_. Similarly, those who exceeded 96% HR_Max_ experienced impaired RHI after SBFF, while it was unchanged for those with lower peak HR. Lastly, LVEDD was only reduced after SBFFP within the group exceeding 101.7% HR_Max_. Indeed, greater cardiovascular strain may beget greater acute cardiovascular impairment. Further research with greater statistical power is needed to confirm this, and examine the effect of fitness on the relationship between cardiovascular strain and impairment.

### 4.1 Limitations

The intensity of the SBFFP was not matched between participants. Instead, participants were instructed to complete as many circuits of work as possible at an ‘urgent’ pace. This created an individualized protocol, allowing each participant to approach or reach maximum exertion over time, regardless of fitness. This is important because self‐paced, initially sub‐maximal work rates are reflective of typical fireground intensity (Langford et al., 2023). However, this design did not allow fitness to be isolated as a predictor of cardiovascular strain. Furthermore, this protocol investigated the cardiovascular strain resulting from a single prolonged work bout. Repeated work bouts are common in both structural and shipboard firefighting. Therefore, further research should investigate the effect of fitness and repeated work bouts on cardiovascular responses to shipboard firefighting.

### 4.2 Conclusion

Similar to structural firefighting, simulated shipboard firefighting results in cardiovascular impairment even in apparently healthy individuals. In this study, we have shown that firefighting activities, which result in hyperthermia and near‐maximum HR, may lead to acute cardiovascular impairment that persists during recovery. Altogether, the cardiac diastolic dysfunction, impaired microvascular function, and reduced myocardial reserve reported here illustrate a scenario of systemic cardiovascular impairment that could partially explain the prevalence of on‐duty cardiac events among structural firefighters.

## AUTHOR CONTRIBUTIONS

David Hostler was responsible for conception and design of the work, with contributions from all authors. Daniel K. Sweet and Elizabeth M. Lavoie led data acquisition. Daniel K. Sweet drafted the manuscript and performed statistical analyses. Hayden W. Hess, Stuart Inglis, Brian Monaco, J. Luke Pryor, and Steven E. Reis provided guidance from methodologic development through manuscript revision. All authors approved the final version of the manuscript and agree to be accountable for all aspects of the work in ensuring that questions related to the accuracy or integrity of any part of the work are appropriately investigated and resolved. All persons designated as authors qualify for authorship, and all those who qualify for authorship are listed.

## CONFLICT OF INTEREST

None declared.

## Data Availability

Deidentified data are available upon reasonable request after IRB approval.
